# Optimal Estimation of Quantum Coherence by Bell State Measurement: A Case Study

**DOI:** 10.3390/e25101459

**Published:** 2023-10-17

**Authors:** Yuan Yuan, Xufeng Huang, Yueping Niu, Shangqing Gong

**Affiliations:** 1 School of Physics, East China University of Science and Technology, Shanghai 200237, China; 2 Shanghai Engineering Research Center of Hierarchical Nanomaterials, Shanghai 200237, China; 3 Shanghai Frontiers Science Center of Optogenetic Techniques for Cell Metabolism, Shanghai 200237, China

**Keywords:** quantum coherence, Bell state measurement, maximum likelihood estimation, quantum measurement

## Abstract

Quantum coherence is the most distinguished feature of quantum mechanics. As an important resource, it is widely applied to quantum information technologies, including quantum algorithms, quantum computation, quantum key distribution, and quantum metrology, so it is important to develop tools for efficient estimation of the coherence. Bell state measurement plays an important role in quantum information processing. In particular, it can also, as a two-copy collective measurement, directly measure the quantum coherence of an unknown quantum state in the experiment, and does not need any optimization procedures, feedback, or complex mathematical calculations. In this paper, we analyze the performance of estimating quantum coherence with Bell state measurement for a qubit case from the perspective of semiparametric estimation and single-parameter estimation. The numerical results show that Bell state measurement is the optimal measurement for estimating several frequently-used coherence quantifiers, and it has been demonstrated in the perspective of the quantum limit of semiparametric estimation and Fisher information.

## 1. Introduction

Quantum entanglement is one of the most subtle and most widely exploited effects in the quantum world, which plays a central role as an essential resource in quantum information science [1]. Bell states are special cases of bipartite maximally entangled states [2] and have been widely studied and used. Bell state measurement (BSM) is itself entangling: it is the projection onto the basis of Bell states and plays crucial roles in many quantum information tasks such as quantum teleportation [3,4,5], quantum key distribution [6,7], quantum dense coding [8,9], entanglement swapping [10,11]. In recent years, to study quantum teleportation for complex quantum systems and develop high-efficiency quantum information networks [12,13], a high-dimensional Bell state measurement has been realized experimentally [14,15,16]. BSM mainly determines the efficiency of these quantum communication tasks; Therefore, BSM is of great significance in quantum information technology. Furthermore, BSM can also as a collective measurement performed on the two-copy state to achieve the direct measurement of quantum coherence [17].

Quantum coherence is a fundamental feature of the quantum system, which describes the superposition of quantum states. Since it has been quantified within the framework of resource theory, quantum coherence is a fundamental resource and is also closely related to quantum entanglement [18,19,20], such as detecting coherence to witness multipartite entanglement and the conversion between coherence and quantum correlations in bipartite and multipartite systems [21,22,23]. Like quantum entanglement, the application of quantum coherence in quantum information technology has also been widely studied [24]. In quantum algorithms, the success probability of quantum algorithms is also related to the coherence of quantum states [25]. In quantum channel discrimination, using the coherent state as the probe state can improve the success probability of discrimination [26]; in quantum communication, using coherence helps to quantify key rate in quantum key distribution [27], as well as applying it to quantum computation and quantum metrology [28]. Being a fundamental property of quantum systems, coherence plays an important role in nanoscale physics [29], transport theory [30], biological systems [31], and quantum thermodynamics [32]. Especially in quantum thermodynamics, coherence enables power outputs that greatly exceed the power of stochastic engines [33]. In addition, quantum coherence is also related to quantum foundations, such as the study of the wave-particle duality [34,35] and quantum uncertainty [36]; using coherence characterizes the wave nature of the quantons, and coherence can be interpreted as a manifestation of quantum uncertainty.

Having identified quantum coherence as a valuable feature of quantum systems and a fundamental quantum resource, it is important to develop methods for its rigorous quantification. While quantum coherence has been extensively studied and various quantifiers have been proposed [24], how to efficiently estimate coherence experimentally is still a challenge, which also limits the application of coherence in quantum information processing. Clearly, one can perform quantum state tomography and then use the derived density matrix of state to calculate the amount of coherence, but this method contains redundant information because the quantifier of coherence is not always related to complete information about the systems. Actually, there are also some strategies to estimate the coherence of unknown quantum states [37,38,39,40,41,42,43], such as using interference fringes [37], spectrum estimation [38,39], and using a numerical optimization algorithm to estimate the coherence based on limited experimental data [40]. Some of these methods only apply to the specific quantifiers of coherence, and most theoretical methods estimate upper and lower bounds for the coherence of unknown quantum states by mathematical calculations and numerical optimizations, which are not very accurate. However, BSM can, as a two-copy collective measurement, directly measure the quantum coherence of an unknown quantum state [17], and does not need any optimization procedures, feedback, or complex mathematical calculations. In this work, we adopt semiparametric estimation theory and single-parameter estimation to analyze the performance of estimating quantum coherence with Bell state measurement for the qubit, respectively. We find that BSM is the optimal measurement for estimating several frequently used coherence quantifiers, and we demonstrate it from the perspective of semiparametric estimation and Fisher information. In the framework of semiparametric estimation theory [44], the mean-square error of the estimation reaches the quantum limit. In the framework of single-parameter estimation, we use maximum likelihood estimation to process data, and the mean-square error of the estimation can reach the Cramér–Rao bound.

## 2. Estimation of Quantum Coherence with Quantum Semiparametric Estimation

Collective measurement scheme estimates the coherence of a quantum state ρ by performing measurements on two-copy state ρ⊗ρ, which provides a simple method to measure coherence, because the entire experiment can be performed in a single measurement setup, and other estimation methods (e.g., tomography) typically require to change the measurement setup many times. For single-qubit states $\rho =\frac{1}{2}\left(I+r\cdot \sigma \right)$ with Bloch vector $r=(r_{x},r_{y},r_{z})$, a collective measurement in the maximally entangled basis $|\psi^{\pm }\rangle =(|01\rangle \pm |10\rangle )/\sqrt{2}$ and $|\varphi^{\pm }\rangle =(|00\rangle \pm |11\rangle )/\sqrt{2}$, i.e., BSM, which consists of four POVM elements

(1) $$\begin{array}{cc} E_{1} & =|\psi^{+}\rangle \langle \psi^{+}|,\;\;\;\;\;E_{2}=|\psi^{-}\rangle \langle \psi^{-}|,\\ E_{3} & =|\varphi^{+}\rangle \langle \varphi^{+}|,\;\;\;\;\;E_{4}=|\varphi^{-}\rangle \langle \varphi^{-}|, \end{array}$$

performed on a two-copy state ρ⊗ρ. The corresponding outcome probabilities $p_{i}=\mathrm{Tr}\left[E_{i}\rho ⊗\rho \right]$, and are denoted as

(2) $$\begin{array}{cc} p_{1} & =\mathrm{Tr}\left[E_{1}\rho ⊗\rho \right]=\frac{1}{4}\left(1+r_{x}^{2}+r_{y}^{2}-r_{z}^{2}\right),\\ p_{2} & =\mathrm{Tr}\left[E_{2}\rho ⊗\rho \right]=\frac{1}{4}\left(1-r_{x}^{2}-r_{y}^{2}-r_{z}^{2}\right),\\ p_{3} & =\mathrm{Tr}\left[E_{3}\rho ⊗\rho \right]=\frac{1}{4}\left(1+r_{x}^{2}-r_{y}^{2}+r_{z}^{2}\right),\\ p_{4} & =\mathrm{Tr}\left[E_{4}\rho ⊗\rho \right]=\frac{1}{4}\left(1-r_{x}^{2}+r_{y}^{2}+r_{z}^{2}\right). \end{array}$$

For an arbitrary coherence, measure $C_{x}$ of the qubit can be expressed as simple functions of $p_{i}$. For example, the ${𝓁}_{1}$-norm of coherence $C_{{𝓁}_{1}}$ and the relative entropy of coherence $C_{r}$ are defined as [45]

(3) $$\begin{array}{cc} C_{{𝓁}_{1}}\left(\rho \right) & =\sum_{i\neq j}^{i,j}\left|\rho_{ij}\right|, \end{array}$$

(4) $$\begin{array}{cc} C_{r}\left(\rho \right) & =S\left(\rho_{diag}\right)-S\left(\rho \right), \end{array}$$

where $S\left(\rho \right)=-\mathrm{Tr}\left[\rho \log_{2}\rho \right]$ is the von Neumann entropy, $\rho_{diag}=\sum_{i}\left|i\rangle \langle i|\rho |i\rangle \langle i\right|$, and we consider coherence with respect to the basis {|i〉}. For single-qubit states $r=(r_{x},r_{y},r_{z})$, both quantities can be expressed as [46]

(5) $$\begin{array}{cc} C_{{𝓁}_{1}}\left(\rho \right) & =\sqrt{r_{x}^{2}+r_{y}^{2}}=\sqrt{2(p_{1}-p_{2})}, \end{array}$$

(6) $$\begin{array}{cc} C_{r}\left(\rho \right) & =h\left(\frac{1+\left|r_{z}\right|}{2}\right)-h\left(\frac{1+r}{2}\right), \end{array}$$

with the binary entropy $h\left(x\right)=-x\log_{2}x-\left(1-x\right)\log_{2}\left(1-x\right)$ and the Bloch vector length $r={\left(r_{x}^{2}+r_{y}^{2}+r_{z}^{2}\right)}^{1/2}$. According to Equation (2), $|r_{z}|$ and *r* can be expressed as $|r_{z}|=\sqrt{2\left(p_{3}+p_{4}\right)-1}$ and $r=\sqrt{1-4p_{2}}$, respectively. Therefore, directly substituting the probability obtained from the experiment into Equations (5) and (6) can directly obtain the value of coherence.

Quantum semiparametric estimation theory is especially relevant to the estimation of a parameter that can be expressed as a function of ρ. We suppose that an experimenter receives *N* quantum objects and estimates a parameter β as a function of ρ, and we let β=Tr(ρY) and *Y* be given observables. The theory can provide the fundamental limit to the precision of estimation for any measurement. For any measurement, the mean-square error E of the estimation has a quantum limit given by [44]

(7) $$E\geq \frac{1}{N}Tr\rho {\left(Y-\beta \right)}^{2}.$$

Taking the estimating of the ${𝓁}_{1}$-norm of coherence as an example, according to Equation (5), for simplicity, we set $C_{{𝓁}_{1}}^{2}$ as the parameter β, and *Y* is expressed as $Y=2(E_{1}-E_{2})$. Here, ρ is two-copy state. Using Equation (7), we can calculate the quantum limit of the mean-square error of estimating $C_{{𝓁}_{1}}^{2}$, and finally use the error transfer formula to obtain the bound of estimating $C_{{𝓁}_{1}}$.

**Figure 1:**
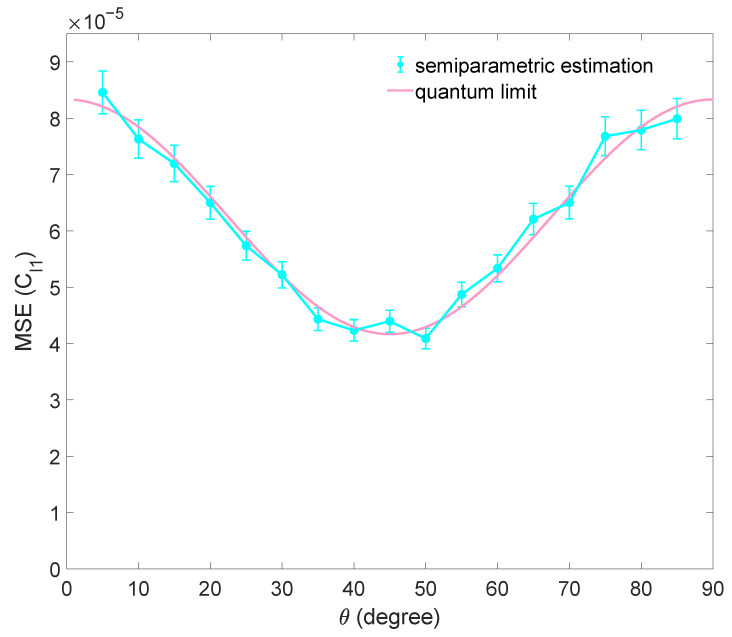
The result of numerical simulation for estimating $C_{{𝓁}_{1}}$ with BSM in semiparametric estimation. The mean squared error (MSE) for estimating $C_{{𝓁}_{1}}$ of a family of qubit states |Ψ〉 is shown. The sample size is N=6000. Each data point is the average of 1000 repetitions, and the error bars denote the standard deviation. The quantum limit of the estimation is shown as a red curve.

Next, we numerically show the performance of estimating several frequently used coherence quantifiers $C_{x}$ with Bell state measurement from the perspective of semiparametric estimation. We consider a single-qubit state |Ψ〉=sinθ|0〉+cosθ|1〉, with θ ranging from 0 to π/2. Bell state measurement is performed on a two-copy state $(|\Psi \rangle {\langle \Psi |)}^{⊗2}$. The sample size *N* is set to 6000. The estimation precision quantifier is the mean squared error (MSE)

(8) $$\Delta^{2}C_{x}^{\mathrm{est}}:=E\left[{\left(C_{x}^{\mathrm{est}}-C_{x}\right)}^{2}\right],$$

where $C_{x}$ is the actual coherence value for a specific quantifier `x’, and $C_{x}^{\mathrm{est}}$ is the estimated value. Firstly, the BSM is performed *N* times on *N* identically prepared states, and outcome *j* occurs $n_{j}$ times. We simulate and generate experimental data $D=\{n_{1},n_{2},n_{3},n_{4}\}$; here, $N=n_{1}+n_{2}+n_{3}+n_{4}$. Next, probabilities $P_{j}=\frac{n_{j}}{N}$ are calculated, and we substitute the probability obtained from simulated experimental data into Equation (5) to estimate the ${𝓁}_{1}$-norm of coherence $C_{{𝓁}_{1}}$. The result of the numerical simulation is shown in Figure 1. As we see from the data shown in Figure 1, the mean squared errors reach the quantum limit obtained by Equation (7).

**Figure 2:**
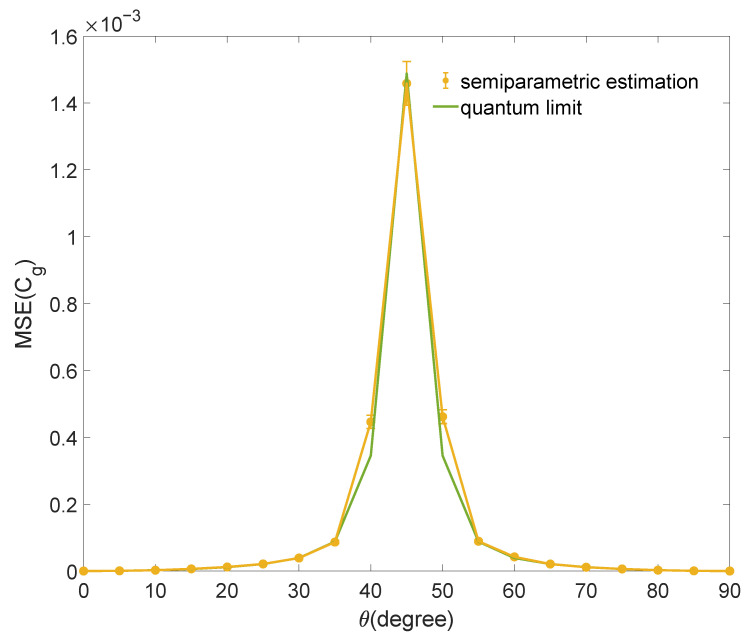
The result of numerical simulation for estimating $C_{g}$ with the $C_{{𝓁}_{1}}$ estimated by semiparametric estimation. The mean squared error (MSE) for estimating $C_{g}$ of a family of qubit states |Ψ〉 is shown. The sample size is N=6000. Each data point is the average of 1000 repetitions, and the error bars denote the standard deviation. The quantum limit of the estimation is shown as a green curve.

Similarly, we also estimate other coherence quantifiers for the qubit states |Ψ〉. In the qubit case, the geometric coherence $C_{g}$ is defined as [24]

(9) $$C_{g}\left(\rho \right)=\frac{1}{2}\left(1-\sqrt{1-4|\rho_{01}{|}^{2}}\right)=\frac{1}{2}\left(1-\sqrt{1-C_{{𝓁}_{1}}^{2}}\right),$$

where $\rho_{01}=\langle 0|\rho |1\rangle$ is the off-diagonal element of ρ in the incoherent basis, and note that for all single-qubit states, we have $C_{{𝓁}_{1}}=2\left|\rho_{01}\right|$. We substitute the $C_{{𝓁}_{1}}$ estimated by semiparametric estimation into function expression Equation (9) to estimate $C_{g}$ and calculate the mean squared error. The bound of estimating $C_{g}$ is obtained by the bound of estimating $C_{{𝓁}_{1}}$ and the error transfer formula. The numerical simulation result for estimating $C_{g}$ is shown in Figure 2. The mean squared errors of numerical simulation reach the quantum limit.

In addition, the coherence of formation $C_{f}$ also can be evaluated exactly. $C_{f}$ in the qubit case is expressed as [24]

(10) $$C_{f}\left(\rho \right)=h\left(\frac{1+\sqrt{1-4|\rho_{01}{|}^{2}}}{2}\right)=h\left(\frac{1+\sqrt{1-C_{{𝓁}_{1}}^{2}}}{2}\right).$$

**Figure 3:**
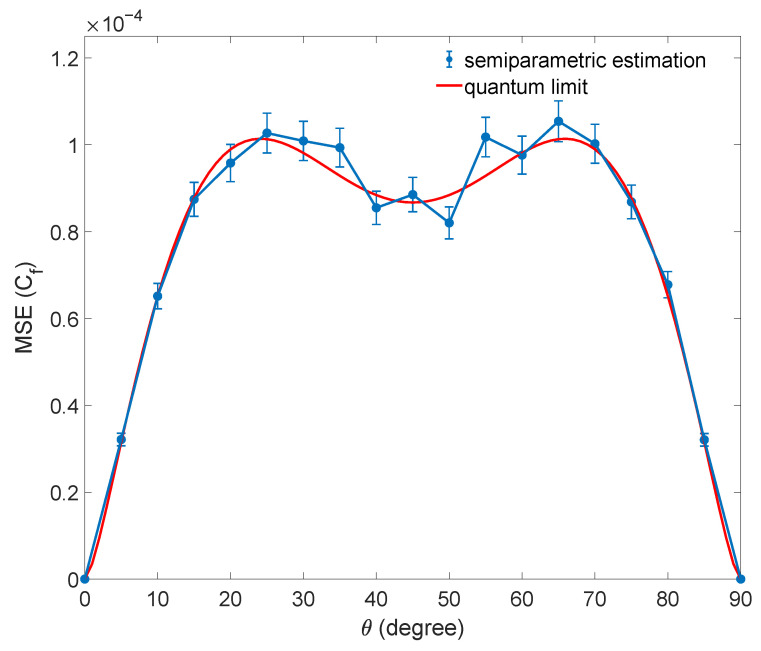
The result of numerical simulation for estimating $C_{f}$ with the $C_{{𝓁}_{1}}$ estimated by semiparametric estimation. The mean squared error (MSE) for estimating $C_{f}$ of a family of qubit states |Ψ〉 is shown. The sample size is N=6000. Each data point is the average of 1000 repetitions, and the error bars denote the standard deviation. The quantum limit of the estimation is shown as a red curve. Only the results of estimating $C_{f}$ are shown here, as these quantities $C_{c}$ and $C_{r}$ are the same as $C_{f}$.

Similarly, we substitute the $C_{{𝓁}_{1}}$ estimated by semiparametric estimation into function expression Equation (10) to estimate $C_{g}$ and calculate the mean squared error. The bound of estimating $C_{g}$ is obtained by the bound of estimating $C_{{𝓁}_{1}}$ and the error transfer formula. The result of the numerical simulation is shown in Figure 3.

Comparing Equation (10) with the geometric coherence of Equation (9), it follows that

(11) $$C_{f}=h\left(1-C_{g}\right),$$

which holds for any single-qubit state. With this relation, we also use the result of $C_{g}$ to calculate $C_{f}$ and the corresponding quantum limit. Another important quantifier is the coherence cost $C_{c}$, which is equal to the coherence of formation $C_{c}=C_{f}$ [24]. When the qubit is a pure state, $|r_{z}|=\sqrt{1-(r_{x}^{2}+r_{y}^{2})}$, Equation (6) is written as $C_{r}=h\left(\frac{1+\sqrt{1-C_{{𝓁}_{1}}^{2}}}{2}\right)$, the relative entropy of coherence $C_{r}$ is equal to Equation (10) of coherence formation. Therefore, the simulation results of these coherence quantifiers are the same as the simulation results of $C_{f}$.

## 3. Estimation of Quantum Coherence with a Single-Parameter Estimation

For the qubit states |Ψ〉=sinθ|0〉+cosθ|1〉 discussed in the simulation above, we can also analyze the performance of estimating coherence $C_{x}$ with Bell state measurement from the perspective of single-parameter estimation.

Firstly, we demonstrate that Bell state measurement is the optimal measurement for estimating several frequently used coherence quantifiers discussed above from the perspective of Fisher information. Fisher information is derived from statistics and used to quantify the estimation accuracy of parameters, such as the Cramér–Rao bound. It plays an important role in quantum information technology, especially in quantum metrology. We recall that quantum Fisher information of parameterized quantum states $\rho =\rho (C_{x})$ is defined as

(12) $${Ƒ}_{C_{x}}=\mathrm{Tr}\rho L_{C_{x}}^{2},$$

where $C_{x}$ is a parameter to be estimated, and $L_{C_{x}}$ is the symmetric logarithmic derivative determined by $\frac{\partial }{\partial C_{x}}\rho =\frac{1}{2}\left(L_{C_{x}}\rho +\rho L_{C_{x}}\right)$. It can be calculated as [47]

(13) $${Ƒ}_{C_{x}}=\sum_{jk}\frac{2}{\lambda_{j}+\lambda_{k}}{\left|\langle j|\frac{\partial }{\partial C_{x}}\rho |k\rangle \right|}^{2}$$

by spectral decomposition $\rho =\Sigma_{j}\lambda_{j}\left|j\rangle \langle j\right|$. Quantum Fisher information sets a fundamental bound to the attainable optimal estimation precision. Using Equation (13), we can obtain the quantum Fisher information of this original state $(|\Psi \rangle {\langle \Psi |)}^{⊗2}$ concerning the parameter $C_{{𝓁}_{1}}$ is ${Ƒ}_{C_{{𝓁}_{1}}}=2/\left(1-C_{{𝓁}_{1}}^{2}\right)$.

We suppose that with a series of measurement operators $\left\{M_{j}\right\}$ independent of the parameter, with $M_{j}\geq 0\forall j$, $\Sigma_{j}M_{j}=I_{d}$, performed on the state $\rho (C_{x})$, the probability distribution is obtained as $p_{j}\left(C_{x}\right)=\mathrm{Tr}\left(\rho \left(C_{x}\right)M_{j}\right)$. Therefore, the measurement-induced Fisher information is defined as

(14) $$F_{C_{x}}=\sum_{j}p_{j}\left(C_{x}\right){\left(\frac{\partial }{\partial C_{x}}\ln p_{j}\left(C_{x}\right)\right)}^{2},$$

which is the classical Fisher information of the measurement-induced probability distribution.

If the estimation of parameter $C_{x}$ is an unbiased estimator, the mean squared error is lower bounded by the Cramér–Rao bound [48]:

(15) $$N\Delta^{2}C_{x}\geq \frac{1}{F_{C_{x}}}\geq \frac{1}{{Ƒ}_{C_{x}}}.$$

Quantum Fisher information (QFI) is the largest Fisher information (FI) upon optimizing the choice of measurement, i.e., ${Ƒ}_{C_{x}}:=\max_{\left\{M_{j}\right\}}F_{C_{x}}$. It can be easily evaluated that classical Fisher information of estimating parameter $C_{{𝓁}_{1}}$ with BSM according to Equation (14) is

(16) $$F_{C_{{𝓁}_{1}}}=\frac{2}{1-C_{{𝓁}_{1}}^{2}}.$$

BSM-induced classical Fisher information reaches quantum Fisher information ($F_{C_{{𝓁}_{1}}}={Ƒ}_{C_{{𝓁}_{1}}}$), which implies BSM is optimal.

Similarly, by utilizing the functional relations between different quantifiers, the Fisher information and quantum Fisher information for $C_{g}$ and $C_{f}$ can be expressed as

(17) $$\begin{array}{cc} F_{C_{g}}={Ƒ}_{C_{g}} & =\frac{2}{C_{g}-C_{g}^{2}}, \end{array}$$

(18) $$\begin{array}{cc} F_{C_{f}}={Ƒ}_{C_{f}} & ={\left(\frac{1}{\log_{2}\frac{1-C_{g}}{C_{g}}}\right)}^{2}\frac{2}{C_{g}-C_{g}^{2}}, \end{array}$$

where Equation (17)obtained using $C_{f}$ is a simple function of $C_{g}$ in the qubit detailed as Equation (11).

Next, we use numerical simulation to verify Equation (15). In the numerical simulation, maximum likelihood estimation (MLE) is used to process data. Maximum likelihood estimation is a method of estimating the parameters of an assumed probability distribution given some observed data. We consider measurement of the coherence of state ρ=|Ψ〉〈Ψ| with BSM ${\left\{E_{j}\right\}}_{j=1}^{m}$ composed of m=4 outcomes, and the probability of obtaining outcome *j* is $p_{j}=\mathrm{Tr}\left[E_{j}\rho ⊗\rho \right]$. The quantifier of coherence $C_{x}$ to be estimated is then contained in the probability $p_{j}\left(C_{x}\right)$ because $C_{x}$ is related to some parameters of the state ρ. If the BSM is performed *N* times on *N* identically prepared quantum systems, outcome *j* occurs $n_{j}$ times with $\sum_{j}n_{j}=N$. Now, we aim to infer the quantifier of coherence $C_{x}$ from the measurement data $D=\{n_{1},n_{2},...,n_{m}\}$. Maximum likelihood estimation searches for the $C_{x}^{ML}$ that maximizes the likelihood function [48] are

(19) $$C_{x}^{ML}:=\arg\max_{C_{x}}L\left(D|C_{x}\right),\;\;\;with\;\;\;L\left(D|C_{x}\right)=\prod_{j}p_{j}^{n_{j}},$$

where $L(D|C_{x})$ is the likelihood of observing the data *D*. The parameter we solve is used to maximize the value of the Likelihood function, which can be solved by derivation in mathematical problems. In practice, it is more convenient to work with the log-likelihood function, because the monotonically increasing nature of logarithmic functions ensures that they do not change the extreme points, and taking the logarithm facilitates our subsequent derivation.

We take $C_{{𝓁}_{1}}$ as an example to demonstrate this method. We cnsider a single-qubit state |Ψ〉=sinθ|0〉+cosθ|1〉 and BSM performed on the two-copy state. The corresponding outcome probabilities $p_{i}=\mathrm{Tr}\left[E_{i}(|\Psi \rangle {\langle \Psi |)}^{⊗2}\right]$ are expressed as

(20) $$\begin{array}{cc} p_{1} & =\frac{1}{2}C_{{𝓁}_{1}}^{2},\;\;\;\;\;p_{2}=0,\\ p_{3} & =\frac{1}{2},\;\;\;\;\;p_{4}=\frac{1}{2}-\frac{1}{2}C_{{𝓁}_{1}}^{2}, \end{array}$$

where $C_{{𝓁}_{1}}=\sin2\theta$. SWe suppose BSM is performed *N* times on *N* identically prepared states, and the measurement data $D=\{n_{1},n_{2},n_{3},n_{4}\}$. According to Equation (19), we write the likelihood function as

(21) $$L\left(n_{1},n_{2},n_{3},n_{4};C_{{𝓁}_{1}}\right)={\left(\frac{1}{2}C_{{𝓁}_{1}}^{2}\right)}^{n_{1}}{\left(\frac{1}{2}\right)}^{n_{3}}{\left(\frac{1}{2}-\frac{1}{2}C_{{𝓁}_{1}}^{2}\right)}^{n_{4}}.$$

We take the logarithm of Equation (21),

(22) $$\log L\left(n_{1},n_{2},n_{3},n_{4};C_{{𝓁}_{1}}\right)=n_{1}\log\left(\frac{1}{2}C_{{𝓁}_{1}}^{2}\right)+n_{3}\log\frac{1}{2}+n_{4}\log\left(\frac{1}{2}-\frac{1}{2}C_{{𝓁}_{1}}^{2}\right),$$

take the derivative of the above equation, let the derivative function equal to zero, and solve $C_{{𝓁}_{1}}^{ML}$ as

(23) $$C_{{𝓁}_{1}}^{ML}=\sqrt{\frac{n_{1}}{n_{1}+n_{4}}}.$$

**Figure 4:**
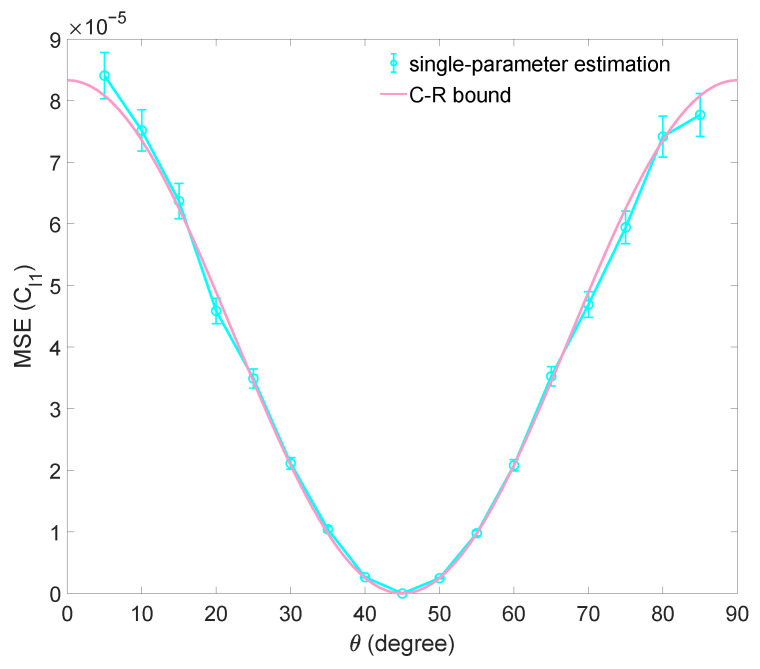
The result of numerical simulation for estimating $C_{{𝓁}_{1}}$ with BSM in single-parameter estimation. The mean squared error (MSE) for estimating $C_{{𝓁}_{1}}$ of a family of qubit states |Ψ〉 using maximum likelihood estimation is shown. The sample size is N=6000. Each data point is the average of 1000 repetitions, and the error bars denote the standard deviation. The Cramér–Rao bound is shown as a red curve.

For the numerical simulation, the coherence of single-qubit states |Ψ〉 with θ ranging from 0 to π/2 are estimated. The sample size *N* is chosen to be 6000. We substitute the simulated measurement data $D=\{n_{1},n_{2},n_{3},n_{4}\}$ into Equation (23) to obtain the $C_{{𝓁}_{1}}^{ML}$ and calculate the mean squared error. The result of the numerical simulation is shown in Figure 4. The mean squared error achieved by the maximum likelihood estimation reaches the Cramér–Rao bound $1/(N{Ƒ}_{C_{{𝓁}_{1}}})$.

**Figure 5:**
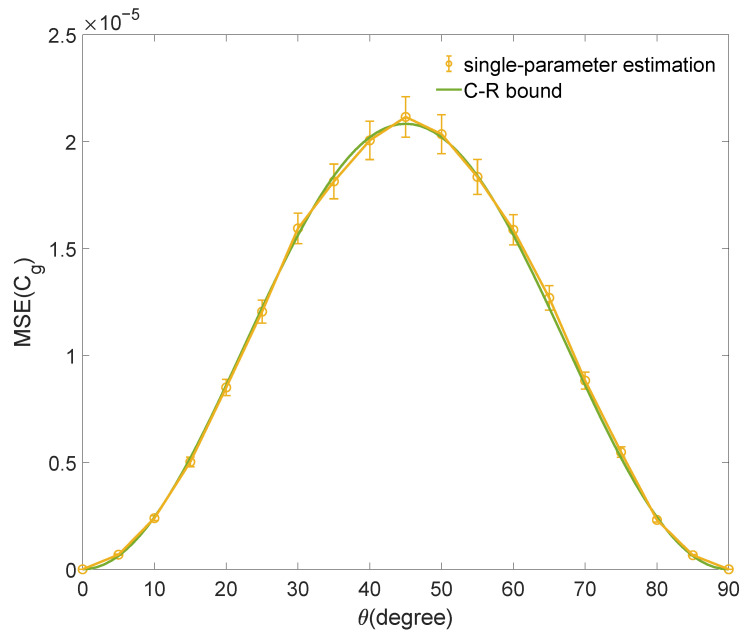
The result of numerical simulation for estimating $C_{g}$ with BSM in single-parameter estimation. The mean squared error (MSE) for estimating $C_{g}$ of a family of qubit states |Ψ〉 using maximum likelihood estimation is shown. The sample size is N=6000. Each data point is the average of 1000 repetitions, and the error bars denote the standard deviation. The Cramér–Rao bound is shown as a green curve.

**Figure 6:**
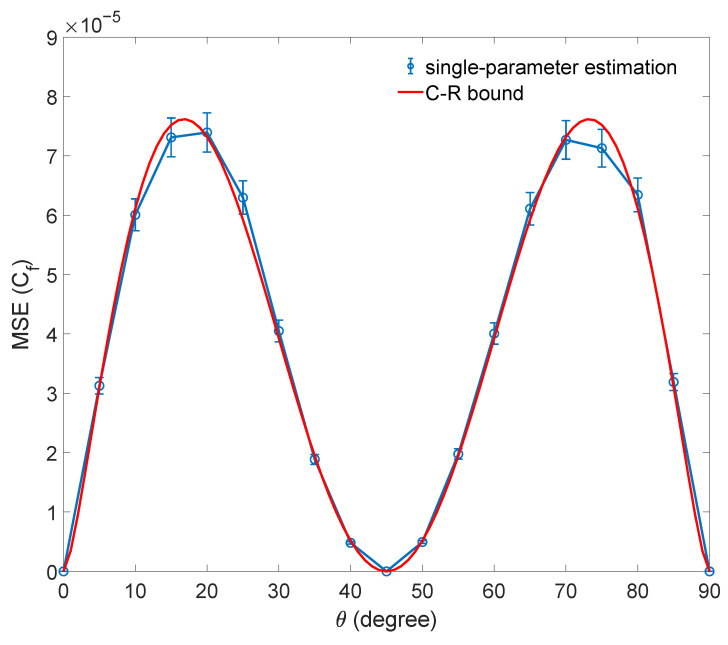
The result of numerical simulation for estimating $C_{f}$ with BSM in single-parameter estimation. The mean squared error (MSE) for estimating $C_{f}$ of a family of qubit states |Ψ〉 using maximum likelihood estimation is shown. The sample size is N=6000. Each data point is the average of 1000 repetitions, and the error bars denote the standard deviation. The Cramér–Rao bound is shown as a red curve. The mean squared error (MSE) for estimating quantities $C_{c}$ and $C_{r}$ are the same as $C_{f}$.

Similarly, we can also use maximum likelihood to estimate geometric coherence and coherence of formation. According to Equation (19) and the maximum likelihood estimation steps, the estimated value of geometric coherence $C_{g}^{ML}$ is

(24) $$C_{g}^{ML}=\frac{1}{2}-\frac{1}{2}\sqrt{\frac{n_{4}}{n_{1}+n_{4}}},$$

which also can be obtained by the relation $C_{g}=\frac{1}{2}\left(1-\sqrt{1-C_{{𝓁}_{1}}^{2}}\right)$. The numerical simulation result for estimating $C_{g}$ is shown in Figure 5. The estimated value of geometric coherence $C_{f}^{ML}$ can be obtained by relation Equation (11). The result of the numerical simulation is shown in Figure 6. Using the data processing method of the maximum likelihood estimation makes the mean squared error (MSE) for estimating $C_{g}$ and $C_{f}$ reach the Cramér–Rao bounds. Although in the framework of single-parameter estimation, BSM is also optimal for estimating coherence of the state |Ψ〉, it is worth mentioning that collective measurements are unnecessary in general for single-parameter estimation.

## 4. Conclusions

Quantum coherence is the most distinguished feature of quantum mechanics and plays a critical role in emerging quantum technologies. While different quantifiers of coherence have been proposed in the literature, their efficient estimation in today’s experiments remains a challenge. BSM as a collective measurement performed on the two-copy state can achieve the direct measurement for any coherence quantifier of a qubit. In this work, we analyze the performance of estimating quantum coherence with Bell state measurement for a qubit case from the perspective of semiparametric estimation and single-parameter estimation, respectively. Using Bell state measurement, the mean square error of the estimation reaches the quantum limit of semiparametric estimation theory. From the perspective of single-parameter estimation, we use maximum likelihood estimation to process data and the mean-square error can reach the Cramér–Rao bound. Our work provides an alternative method for direct measurement of coherence and highlights the application of BSM in quantum information processing.

## Data Availability

The data presented in this study are available on request from the corresponding author.
